# Conjunctival pyogenic granuloma during pregnancy

**DOI:** 10.5935/0004-2749.20210072

**Published:** 2021

**Authors:** Mehmet Zahid Şekkeli, Melek Banu Hoşal, Elif Öcal, Aylin Heper

**Affiliations:** 1 Department of Ophthalmology, Ankara University Faculty of Medicine, Ankara, Turkey; 2 Department of Pathology, Ankara University Faculty of Medicine, Ankara, Turkey

**Keywords:** Granuloma, pyogenic, Conjunctival Diseases, Pregnancy complications, Case reports, Granuloma piogênico, Doenças da túnica conjuntiva, Complicações na gravidez, Relato de casos

## Abstract

Pyogenic granuloma is a common benign, vascular lesion of the skin and mucosa. Our case was a 34-year-old woman at 28 weeks of gestation. She presented with a complaint of a growing and occasionally bleeding mass on the left lower eyelid conjunctiva for approximately 3 months. On examination, a red, pedunculated fragile lesion on the medial lower eyelid conjunctiva was observed. Ophthalmologic examination revealed that visual acuity was 20/20 in both eyes. Anterior and posterior segment examination was normal bilaterally. The lesion was excised completely under local anesthesia and sent for pathological examination. Histopathological examination revealed multiple vascular structures with small-diameters in the fibrous stroma. Vessels showed strong CD31 expression in immunohistochemical staining. Kaposi sarcoma was excluded due to negative HHV-8 staining. These findings were diagnostic of pyogenic granuloma. The frequency of pyogenic granuloma increases during pregnancy and surgical excision is important in diagnosis and treatment of these patients. This is the first reported case of conjunctival pyogenic granuloma during pregnancy.

## Abstract (pt)

O granuloma piogênico é uma lesão vascular benígna comum da pele e da mucosa. Neste caso, uma mulher de 34 anos com 28 semanas de gestação, apresentou queixa de massa em crescimento e sangramento ocasional na conjuntiva inferior da pálpebra esquerda por cerca de 3 meses. No exame, detectou-se uma lesão frágil, pedunculada vermelha na conjuntiva na porção medial da pálpebra inferior No exame oftalmológico, a acuidade visual foi 20/20 em ambos os olhos. O exame dos segmentos anterior e posterior foi normal bilateralmente. A lesão foi excisada totalmente com anestesia local e enviada para exame patológico. No exame histopatológico, múltiplas estruturas vasculares de pequeno diâmetro foram observadas no estroma fibroso. Os vasos apresentaram forte expressão de CD31 na coloração imuno-histoquímica. O sarcoma de Kaposi foi excluído devido à negatividade da coloração do HHV-8. Esses achados foram diagnósticos para granuloma piogênico. A frequência de granuloma piogênico aumenta durante a gestação e a excisão cirúrgica é importante no diagnóstico e tratamento desses pacientes. Este é o primeiro caso relatado de granuloma piogênico conjuntival ocorrido durante a gestação.

## INTRODUCTION

Pyogenic granuloma (PG) is a benign, vascular lesion of the skin and mucosa. Pyogenic granuloma is most commonly seen on the head, neck, and extremities. Ocular PG may occur on the eyelids, conjunctiva, cornea, or lacrimal sac. It may develop idiopathically or after trauma, ocular surgery, or chalazion. Clinically, PG is a rapidly growing, red-purple, sessile or pedunculated lesion. Bleeding and ulceration are common complications^(1,2)^.

Pyogenic granuloma may be seen at any age, although it is more common in children, adolescents, and pregnant women. The etiology of PG is unclear, but chronic irritation, trauma, pregnancy, and hormonal factors are considered. Hormonal changes during pregnancy or due to the use of oral contraceptives may cause PG by increasing the levels of vascular endothelial growth factor (VEGF) and basic fibroblast growth factor (bFGF)^(3,4)^. Pyogenic granuloma during pregnancy is also called granuloma gravidarum. However, there is no histopathological difference between PG in pregnant and non-pregnant patients^(5)^. Although PG is frequently seen as skin lesions in pregnant women, it may also originate from the mucosa^(3)^.

To our knowledge, PG arising from the eyelid conjunctiva has not been reported in pregnant women. In this study, a case of conjunctival pyogenic granuloma in a pregnant patient is presented.

## CASE REPORT

**Figure 1:**
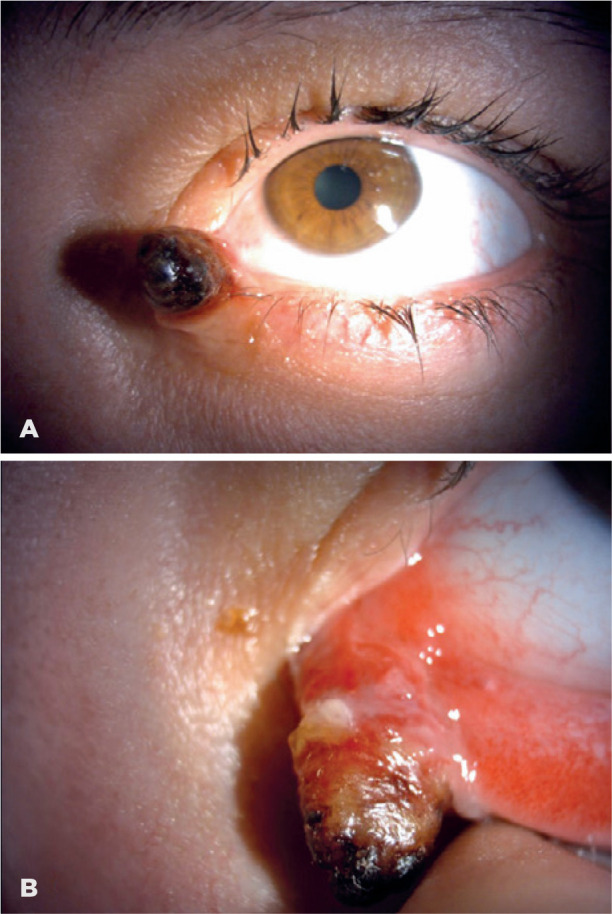
(A and B) A 12 × 5 × 4 mm^3^ necrotic, pedunculated lesion on the lower eyelid conjunctiva of the left eye.

A 34-year-old patient was referred to our clinic with a lesion on her left lower eyelid, which had been present for 3 months. She stated that the mass was growing rapidly and occasionally bleeding. She was at the 28th week of gestation, with no history of trauma, previous ocular surgery, or chalazion. She was not using any topical or systemic drugs. Upon examination, a red, pedunculated, conjunctival mass measuring 12 × 5 × 4 mm^3^ on her left lower eyelid conjunctiva was seen (Figure 1A-B). The lesion was close to the punctum. Ophthalmologic examination revealed that visual acuity was 20/20 and anterior and posterior segment examination was normal in both eyes. Intraocular pressure was 18 mmHg bilaterally.

**Figure 2:**
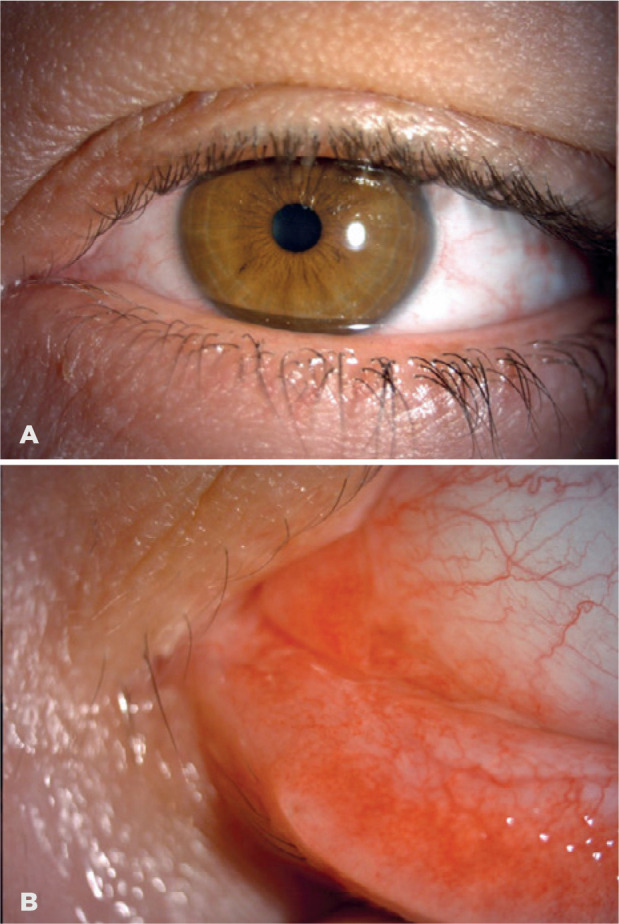
(A and B) Postoperative 1^st^ month appearance.

Total excisional biopsy was planned with local anesthesia. As the lesion was close to the punctum, the punctum and lower canaliculus were protected with lacrimal cannula (26G) and the lesion was removed completely. Bleeding control was achieved with cautery during the operation. Post-operative 1st month appearance is shown in figure 2. No recurrence was observed in the patient for 2 years.

**Figure 3:**
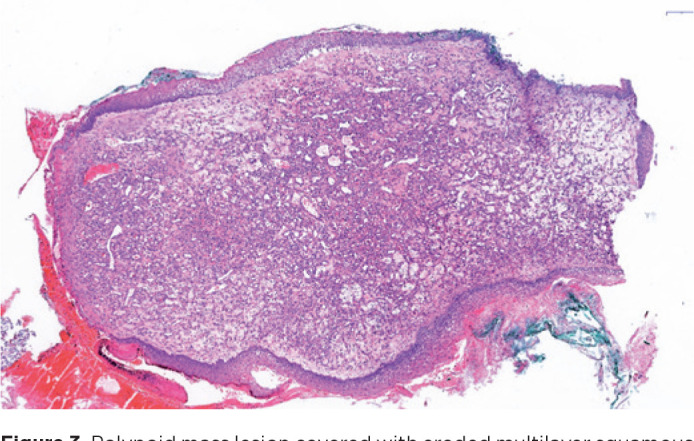
Polypoid mass lesion covered with eroded multilayer squamous epithelium and conjunctival epithelium. The lesion consists of numerous vascular structures with small-diameters, located in the edematous fibrous stroma. H-EX40.

**Figure 4:**
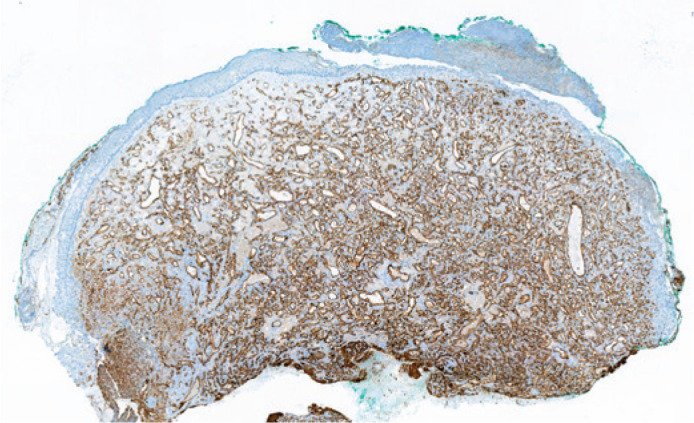
Immunohistochemical examination showing widespread CD31 stroma. H-EX40. expression on the endothelial lining of vascular structures. CD31 x40.

The 12 × 6 × 4 mm^3^ lesion had a nodular appearance on macroscopic examination. The lesion was covered with stratified squamous epithelium and conjunctival mucosa (Figure 3). The underlying fibrous stroma had numerous small vascular structures with a narrow lumen. Some extravasated erythrocytes were present inside the lesion. Ulcerated areas with some neutrophils were seen in the covering epithelium. The vessels showed strong CD31 expression in immunohistochemical staining (Figure 4). Kaposi sarcoma was excluded due to negative HHV-8 staining. These findings were diagnostic for pyogenic granuloma.

## DISCUSSION

Pyogenic granuloma was first described by Poncet and Dor in 1897 as Botryomycosis humaine, and the more commonly used “pyogenic granuloma” was proposed by Hartzell in 1904^(2)^. More recently, the term lobular capillary hemangioma was used for pyogenic granuloma^(3)^. Although the etiology of PG is not clearly defined, chronic irritation, trauma, pregnancy, and hormonal factors are considered. Ocular PG is usually seen after surgical trauma, chalazion, foreign body; it may also be idiopathic. In a report of 100 pyogenic granuloma cases, chalazion was the predisposing factor in 42 cases, ocular surgery in 40 cases, and trauma in 5 cases. No predisposing factor was detected in 13 cases^(1)^. Although PG may be seen at any age, it is more common in children, adolescents, and pregnant women^(3)^.

The frequency of PG due to hormonal changes increases during pregnancy. It is usually seen in the second or third trimesters in 4%-5% of pregnant women. Trauma and female hormones are thought to increase the expression of angiogenic factors, such as bFGF and VEGF, that cause the development of PG^(5)^. It is usually seen on the fingers, palms, and scalp, and also on the mucosa. Mucosal lesions usually occur in the oral cavity or gingival mucosa^(3)^.

The differential diagnosis of PG includes malignant and benign tumors, such as suture granulomas, squamous papillomas, squamous cell carcinoma, and Kaposi’s sarcoma^(4)^. Suture granuloma usually develops in the first week after surgery due to non-absorbable sutures. Topical steroids and surgical excision are used for treatment^(6)^. Squamous papillomas are sessile or pedunculated lesions in pediatric and adult patients. Human papillomavirus (HPV) types 6 and 11 are responsible for the pathogenesis. Surgical excision is used for treatment^(7)^. Squamous cell carcinoma usually occurs in interpalpebral fissures and can also be seen in the eyelid and conjunctiva. It is painless and grows progressively. Ultraviolet light exposure, HPV type 16/18 and acquired immune deficiency syndrome (AIDS) are responsible for the etiology. Surgical excision is commonly used for treatment^(8)^. Kaposi’s sarcoma is seen as a vascular tumor in patients with AIDS. Human herpes virus 8 (HHV-8) is responsible for its etiology. Cryotherapy, chemotherapy, radiotherapy, or antiretroviral therapy can be used for treatment^(9)^.

Different methods such as the application of topical steroids, topical timolol, and silver nitrate; cryotherapy; and surgical excision are used for treating PG. The choice of treatment varies according to the location and severity of the lesion^(10)^. PG seen during pregnancy may regress spontaneously due to the disappearance of hormonal changes at the end of pregnancy^(3)^. The classical treatment of PG is surgical excision^(1-10)^. In this case surgical excision is preferred for histopathological diagnosis and also to exclude malignant lesions.

In conclusion, although the frequency of PG increases during pregnancy, this is the first reported case of PG in the palpebral conjunctiva. Surgical excision of PG is the ideal treatment option because it reduces the risk of recurrence and provides a differential diagnosis of malignant tumors histopathologically.
